# Differential expression of miR-184 in temporal lobe epilepsy patients with and without hippocampal sclerosis – Influence on microglial function

**DOI:** 10.1038/srep33943

**Published:** 2016-09-26

**Authors:** Bénédicte Danis, Marijke van Rikxoort, Anita Kretschmann, Jiong Zhang, Patrice Godard, Lidija Andonovic, Franziska Siegel, Pitt Niehusmann, Etienne Hanon, Daniel Delev, Marec von Lehe, Rafal M. Kaminski, Alexander Pfeifer, Patrik Foerch

**Affiliations:** 1 UCB Pharma S. A., Chemin du Foriest, B - 1420 Braine-l’Alleud, Belgium; 2 Institute of Pharmacology and Toxicology, University of Bonn, Sigmund-Freud-Str. 25, 53127 Bonn, Germany; 3 Thomson Reuters, IP & Science, 5901 Priestly Dr., #200, Carlsbad, CA 92008, USA; 4 Deptartment of Neuro- / Pathology, Oslo University Hospital, Sognsvannsveien 20, 0372 Oslo, Norway; 5 Neurosurgery, University of Bonn, Sigmund-Freud-Str. 25, 53127 Bonn, Germany; 6 Neurosurgery, Universitätsklinikum Knappschaftskrankenhaus Bochum, In der Schornau 23-25, 44892 Bochum, Germany

## Abstract

Epilepsy is one of the most common neurological disorders characterized by recurrent seizures due to neuronal hyperexcitability. Here we compared miRNA expression patterns in mesial temporal lobe epilepsy with and without hippocampal sclerosis (mTLE + HS and mTLE −HS) to investigate the regulatory mechanisms differentiating both patient groups. Whole genome miRNA sequencing in surgically resected hippocampi did not reveal obvious differences in expression profiles between the two groups of patients. However, one microRNA (miR-184) was significantly dysregulated, which was confirmed by qPCR. We observed that overexpression of miR-184 inhibited cytokine release after LPS stimulation in primary microglial cells, while it did not affect the viability of murine primary neurons and primary astrocytes. Pathway analysis revealed that miR-184 is potentially involved in the regulation of inflammatory signal transduction and apoptosis. Dysregulation of some the potential miR-184 target genes was confirmed by qPCR and 3′UTR luciferase reporter assay. The reduced expression of miR-184 observed in patients with mTLE + HS together with its anti-inflammatory effects indicate that miR-184 might be involved in the modulation of inflammatory processes associated with hippocampal sclerosis which warrants further studies elucidating the role of miR-184 in the pathophysiology of mTLE.

Mesial temporal lobe epilepsy (mTLE) is the most common type of epilepsy characterized by recurrent seizures, which arise from the medial structures of the temporal lobe, e. g. hippocampus, parahippocampal gyrus or amygdala^1^,^2^. Up to 50% of the patients suffering from mTLE are pharmacoresistant, experiencing recurrent and damaging seizures^3^. A widely accepted treatment option for pharmacoresistant epilepsy is surgical removal of the epileptogenic area. In recent studies of surgical outcomes up to 65% of the patients were seizure free and up to 28% showed a significant improvement after surgery (for review see^4^). However, epilepsy surgery is an invasive procedure with perioperative risks and potential long term consequences, therefore alternative treatment options are desirable.

The majority of resected hippocampi in mTLE surgery (52%) present hippocampal sclerosis (HS) also called Ammon’s horn sclerosis^5^. HS was already observed by Bouchet in 1825 describing the hippocampi of patients as hardened and atrophied^6^. The typical clinical microscopic hallmark is a characteristic pattern of neuronal loss^7^ with reactive gliosis, mossy fiber sprouting and granule cell dispersion. Depending on the extent and localization of neuronal loss patients can be classified using the modified Wyler’s score^8^ with 0 defining tissue without HS and Wyler Score IV as definition for severe HS. Nowadays, HS can be diagnosed using MRI, the typical feature is unilateral volume loss and increased signal intensity on T2-weigthed images^9^.

Whether HS is a cause or consequence of seizures or contributes to the progression of mTLE is still a matter of debate. However, clinical observations and experimental evidence in animal models highlight brain inflammation as a common factor in mTLE^10^. Uncontrolled seizures, damaged blood-brain barrier (BBB) and persistent inflammation may contribute to the development of chronic inflammation that drives the progression of mTLE + HS (for review see refs 10,11). A better understanding of the molecular events leading to HS might provide insights into the pathological mechanisms and potentially lead to the development of new therapies.

MicroRNAs (miRNAs) are a class of small endogenous non-coding RNAs (~23nt) that regulate gene expression at the post-transcriptional level. MiRNAs bind to a partially complementary sequence of the target mRNA and reduce protein production by blocking translation or inducing mRNA degradation (for review see^12^,^13^). To date over 1800 miRNAs are described in the human genome (miRBase release 21) and are estimated to modulate the levels of at least one third of protein coding messenger RNAs^14^. MiRNAs have been shown to play important roles in different neurodegenerative diseases including epilepsy^15^,^16^. Dysregulation of miRNA expression was described in several animal models of mTLE and few reports address this topic using resected hippocampus from mTLE patients along with work describing the expression pattern of individual miRNAs in human epilepsy^17^,^18^,^19^,^20^,^21^.

To identify molecular differences between mTLE patients with HS and patients without HS we performed RNA deep sequencing analysis investigating genome-wide miRNA expression patterns in human hippocampal samples resected during surgery from epilepsy patients. To our knowledge, this is the first miRNA sequencing effort comparing mTLE + HS vs. mTLE -HS samples. Our aim was to understand if miRNAs contribute to the more pronounced neuronal death or inflammatory responses observed in mTLE + HS patients. Deep sequencing revealed only minor differences in the global miRNA expression profiles between the two patient groups. We identified one miRNA (miR-184) that was differentially expressed. To study the potential function of miR-184 we examined the effects of its over-expression in several *in vitro* assays modelling different aspects of epilepsy pathophysiology. We observed that miR-184 over-expression can modulate cytokine release by activated microglial cells but had no effect on astrocyte or neuronal viability. Taken together, we identified a decreased expression of miR-184 in mTLE + HS patients which might contribute to the chronic inflammatory processes and consequently to a more pronounced neuronal death observed in mTLE + HS patients.

## Results

### miRNA profiling in epilepsy patients comparing mTLE + HS versus mTLE −HS patients

We analysed 24 surgical brain samples from patients with mTLE. All patients suffered from pharmacoresistant mTLE and underwent selective hippocampectomy. The biopsy specimens underwent neuropathological evaluation with qualitative assessment of hippocampal cell loss and reactive gliosis. HS sclerosis was diagnosed in 14 cases (mTLE + HS), whereas 10 cases showed no substantial neuronal cell loss in the pyramidal cell layer (mTLE -HS). In the mTLE-HS group astrogliosis was observed in hippocampal tissues obtained from 8 patients (Table 1). Wyler grades^8^ were determined whenever possible for the classification in the mTLE + HS group (Wyler grade III: n = 4; Wyler grade IV n = 6). Two biopsy samples showed a CA1 predominant neuronal cell loss with only mild affection of the other sectors. This pattern of subfield neuronal cell loss is not represented in the Wyler classification, but has been considered as atypical (type 2) HS in the 2013 ILAE-consensus classification of hippocampal sclerosis^22^. In two cases no definite classification (Wyler or ILAE-consensus classification) was possible due to fragmentation of the biopsy sample. However, NeuN-immunohistochemistry revealed a severe segmental neuronal cell loss in both cases, allowing the diagnosis of HS. (Table 1). There were no statistically significant differences between mTLE + HS and mTLE -HS groups regarding age, disease duration (the p-value for the Wilcoxon rank-sum test was greater than 0.05) or gender (the p-value for the exact Fisher’s test was greater than 0.05) (Supplementary Figure S1).

### miRNA-184 is significantly dysregulated in mTLE +HS patients

**Figure 1:**
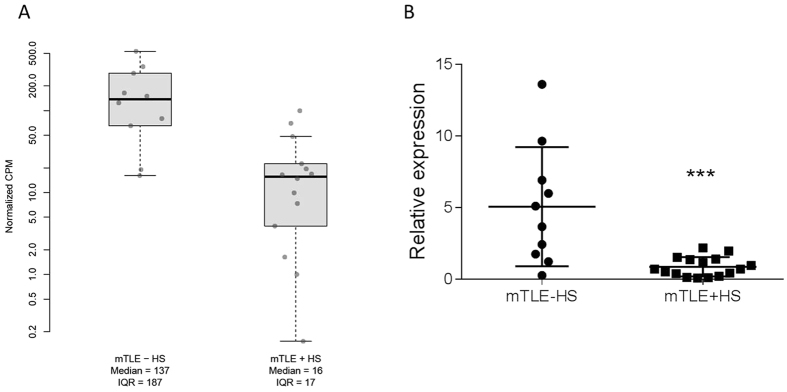
Expression of miR-184 in mTLE patients. **(A)** Comparison of the normalized expression values (CPM: counts-per-million of sequenced RNA) of mir-184 in the 2 groups of patients: mTLE -HS and mTLE + HS. The median and the interquartile range (IQR) of expression values are provided for both groups. **(B)** RT-qPCR validation of miR-184 expression in mTLE -HS and mTLE + HS patients. Data represent the relative gene expression for each patient calculated with qBase + software using miR-125a-5p and miR-191-5p as reference microRNAs. Error bars represent s.d. Statistical analysis was performed using the non-parametric t-test Mann-Whitney (p < 0.001).

**Figure 2:**
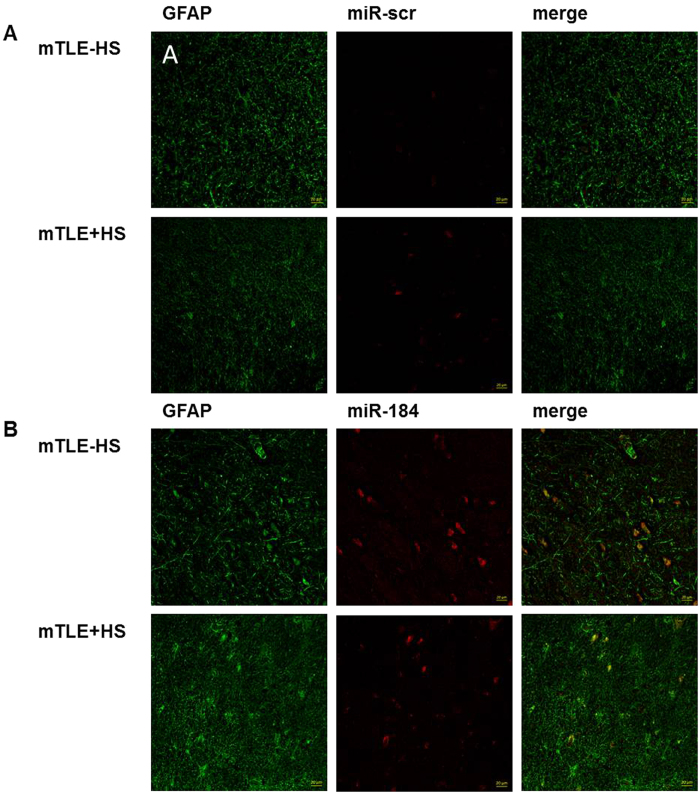
*In situ* hybridisation analysis of miR-184 in mTLE patients. Sections were incubated with probes for miR-184 **(B)**. A scrambled miRNA sequence (miR-scr) **(A)** was used as control. Sections were co-stained with anti-GFAP (green). Representative images are shown (patients: nonHS07 and HS06).

The brain tissue samples were analysed for miRNA expression by deep sequencing and subsequent differential expression analysis. On average, 7.5 million reads were sequenced by sample leading to the identification of 894 known microRNA detected in at least 10 samples. The global miRNA expression pattern of all samples analysed showed only slight differences between mTLE + HS versus mTLE -HS patients highlighted by the heterogeneous distribution of both groups in the PCA plot (Supplementary Figure S2). In addition there was no clear separation of patients with Wyler Score III and Wyler Score IV among the mTLE + HS patients based on the overall miRNA expression pattern. Although overall miRNA signature seems to be similar among the different epilepsy patients, we identified one microRNA (miR-184) that was significantly (FDR < 0.01) down regulated in mTLE + HS patients compared to samples from mTLE -HS patients (Fig. 1A). To verify the results from the RNA sequencing analysis, we investigated the patient samples for expression of miR-184 using RT-qPCR (Fig. 1B). The qPCR results confirmed the downregulation in the mTLE + HS group and revealed a more heterogeneous expression of miR-184 in mTLE -HS patient samples, while in mTLE + HS the expression of miR-184 was consistently low (Fig. 1B). Next we set out to detect miR-184 in the resected tissue by *in situ* hybridization. Although we were able to generate *in situ* data only for two mTLE + HS patients and two mTLE -HS patients, these experiments indicate lower expression levels of miR-184 in mTLE + HS compared to tissue from mTLE -HS (Fig. 2B and Supplementary Figure S3). Taken together, miR-184 was expressed at lower levels in mTLE + HS patients compared to mTLE -HS patients (Figs 1 and 2).

### miR-184 target genes

**Figure 3:**
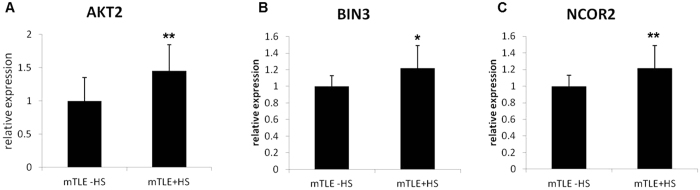
Expression of miR-184 target genes in hippocampal tissue. The expression of selected target genes of miR-184 was investigated by qPCR in samples of mTLE -HS and mTLE + HS patients. Results show means of all patients. Error bars represent s.d. **p < 0.05, *p < 0.1.

Using the MetaCore^TM^ software suite (http:// thomsonreuters.com/site/systems-biology), we found 83 predicted target genes for miR-184, four of them being experimentally validated: AKT2 (PMID: 20409325), NCOR2 (PMID: 22017809), TNFAIP2 (PMID: 21934093) and BIN3 (PMID: 20795863). Supplementary Table S1 shows these genes and also the biological pathways in which they are involved according to MetaCore^TM^. Many of the miR-184 predicted targets belong to pathways related to immune response and apoptosis. We measured the expression of several miR-184 target genes by qPCR in patient samples. Consistent with down-regulation of miR-184 three of the four experimentally validated miR-184 genes, AKT2, BIN-3 and NCOR2 were significantly upregulated in mTLE + HS in comparison with mTLE –HS (Fig. 3A–C). However, several predicted target genes including BCL2L1, CD86, CDK1, GSK3A, NFATC2, PRKCB, PTGS2 and TNFAIP2 were not upregulated in mTLE + HS compared with mTLE -HS (Supplementary Figure S4). To evaluate whether miR-184 might modulate expression of these genes by blocking translation a 3′ UTR luciferase reporter gene assays was conducted for the same genes. Among the 11 genes tested AKT2, BIN3 and PRKCB showed reduced luciferase expression upon co-transfection with miR-184 compared to the control miRNA mimic (miR-src) and untransfected cells while other genes did not (Supplementary Figure S5). These results identified a potential translational regulation of PRKCB by miR-184 as no difference was observed on the mRNA level between the two patient groups (Supplementary Figure S4). The experimentally validated NCOR2 target was not validated in our hands potentially due to the different experimental conditions used (e.g. full length 3′UTR versus short 3′UTR^23^).

These data suggest miR-184 is regulating AKT2, BIN3, and PRKCB expression, genes that are known to be involved in immune response and apoptosis related pathways (Supplementary Table S1). Overall, this implies a potential involvement of miR-184 in the regulation of inflammatory processes and apoptosis. To explore this hypothesis, we investigated the function of miR-184 in a range of cellular assays.

### Influence of miR-184 on microglial activation

**Figure 4:**
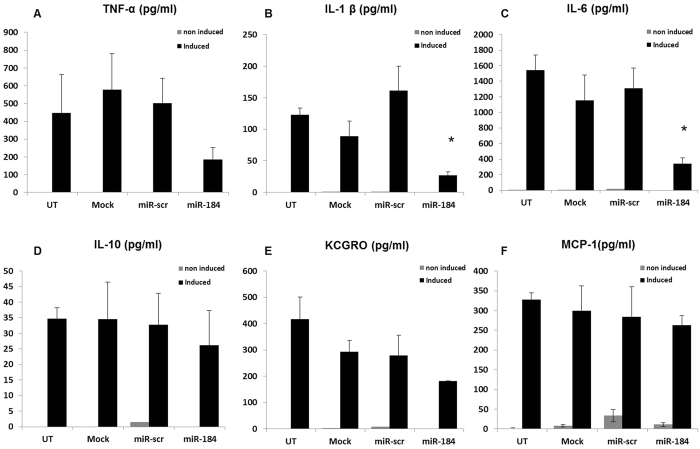
Influence of miR-184 overexpression in primary murine microglial cells. Secreted levels of different cytokines were measured 72 hours after transfection using MSD. **(A)** Tumor Necrosis Factor α (TNF-α), **(B)** Interleukin-1β (IL-1β), **(C**) Interleukin-6 (IL-6), **(D)** Interleukin-10 (IL-10), **(E)** Keratinocyte derived chemokine/growth related oncogene (KC/GRO), **(F)** Monocyte Chemoattractant Protein-1 (MCP-1). Histogramm showing the absolute values of released cytokines with and without prior induction with LPS/IFN-γ. Experiments were performed in n = 4 replicates, measurement n = 2 per condition. Data represent mean +/− s.e. (*p < 0.05).

Inflammatory processes play a role in epilepsy and activated microglial cells can be detected in brains of animal epilepsy models as well as in epilepsy patients^24^,^25^. Therefore, we determined the levels of different pro- and anti-inflammatory cytokines in the supernatant of activated microglial cells upon transfection with miR-184 or control miRNA mimic (miR-scr). Overexpression of miR-184 in stimulated microglial cells resulted in significant changes in cytokine levels while a control miRNA mimic (miR-scr) had no significant effect. Transfection with miR-184 mimic significantly decreased secretion of the pro-inflammatory cytokines Interleukin-6 and Interleukin-1β compared to control miRNA mimic (miR-scr) (Fig. 4B,C), while secretion of Interleukin-10, KCGRO and MCP-1 was not affected by miR-184 (Fig. 4D,F). Levels of TNF-α were lower in miR-184 transfected cells relative to miR-scr transfected cells – albeit not significantly (Fig. 4A). Overexpression of miR-184 was confirmed by qPCR (Supplementary Figure S6A).

### Influence of miR-184 overexpression on primary neurons

**Figure 5:**
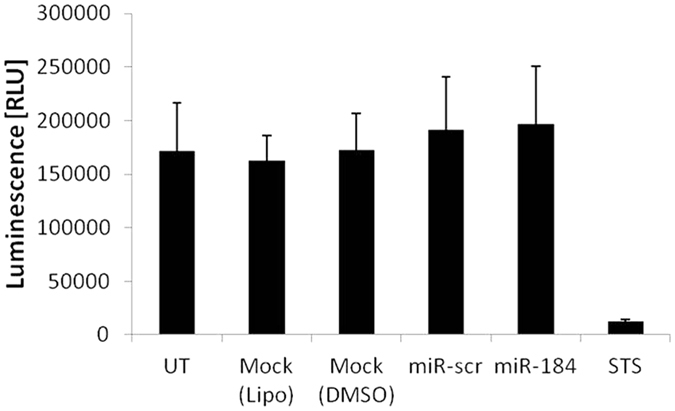
Influence of miR-184 on neuronal viability. Primary murine neurons were transfected with miR-184 mimic and mir-scr. Cytotoxicity was measured using an ATP-release assay 72 h after transfection. Bar graphs represent the mean of 4 independent experiments +/− s.d. 5 μM Staurosporine (STS) served as positive control for the induction of cytotoxicity.

Hippocampal neuronal death and neuronal loss are common pathologic hallmarks of mTLE^26^ and particularly widespread in patients with HS^7^. To investigate a potential involvement of miR-184 in the more pronounced neuronal death observed in mTLE + HS patients, we investigated the influence of miR-184 on viability of primary neurons. We overexpressed miR-184 in murine primary neurons using miRNA mimic and assessed the cell viability 72 h after transfection using an ATPlite-Assay. Transfection with miR-184 mimics did not affect neuronal viability relative to cells transfected with control miRNA mimic (miR-scr) (Fig. 5). Overexpression levels of miR-184 in primary neurons were confirmed by qPCR (Supplementary Figure S6B).

### Influence of miR-184 on astrogliosis

**Figure 6:**
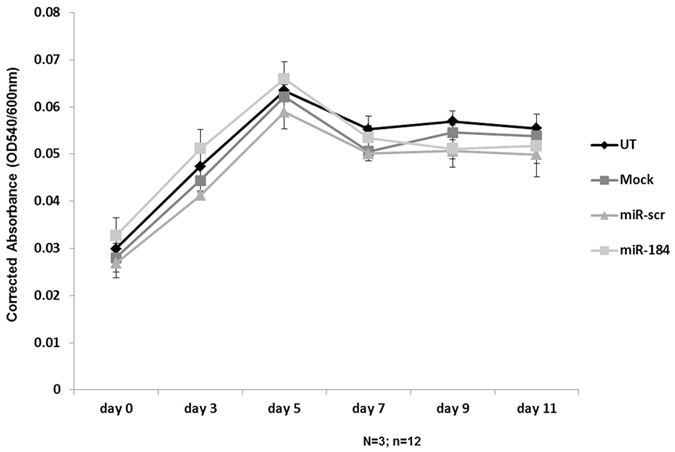
Influence of miR-184 on the viability of astrocytes. Viability of astrocytes was measured by MTT assay from day 0 after transfection until day 11 after transfection. Experiments were performed in n = 3 replicates, measurement n = 4 per condition. Data represent mean +/− s.e.

Reactive astrocytosis is a hallmark of epilepsy and has been described in brain tissue from human epilepsy patients especially in patients with HS^7^,^26^. Similarly, astrogliosis is known to be induced after seizures in experimental models^24^.To investigate the role of miR-184 in astrogliosis and more specifically on glial cell proliferation, we measured cell viability using primary murine astrocytes in presence of miR-184 or a control miRNA mimic (miR-scr). Neither overexpression of miR-184 nor control miRNA mimic had any effect on the cell viability of primary murine astrocytes throughout the entire time course of 11 days (Fig. 6). Overexpression levels of miR-184 in primary astrocytes was verified by qPCR (Supplementary Figure S6C).

## Discussion

Dysregulation of miRNA expression as well as their involvement in the pathophysiology of epilepsy has been described previously and thereby underpinning their potential impact on the disease^15^,^17^. Several miRNA profiling studies in animal models of mTLE and more recently on material from resected hippocampus from mTLE patients were performed along with studies focusing on the functional role of individual miRNAs in human epilepsy^19^,^20^,^21^. Albeit essential to help our understanding of miRNA function in epilepsy, studies relying on human patient samples are more prone to heterogeneity due to several parameters such as gender, age of disease onset, disease duration or medication^27^. In addition availability of suitable control tissue is challenging especially as hippocampi from post mortem tissue cannot be consider as ideal control for surgically resected material from TLE patients.

In our study we aimed to overcome this methodological issue by comparing samples from mTLE patients with HS (mTLE + HS) and mTLE patients without HS (mTLE -HS). With this approach we attempted to unravel the molecular mechanisms involved in the pathological processes of these two mTLE patient groups. We investigated global miRNAs expression using deep sequencing from the hippocampal surgically resected samples comparing mTLE + HS patients to mTLE -HS patients. While the overall expression pattern of all detected miRNAs was not significantly different, we identified one miRNA, miR-184, with significantly reduced expression levels in samples from mTLE + HS patients when compared to mTLE -HS patients. This result was further confirmed by RT-qPCR. Interestingly this observation is consistent with data described previously^19^. Kan *et al*. identified 165 significantly dysregulated miRNAs when comparing three patient groups, mTLE + HS, mTLE -HS and autopsy controls. MiR-184 showed a decreased expression validated by qPCR only in the mTLE + HS group, which is consistent with our data. In contrast a study by Kaalund *et al*., reported an increase in miR-184 expression in the mTLE + HS group when compared to 2 autopsy controls samples^20^. However, this elevation of miR-184 was only seen in the initial microarray data and not confirmed by qPCR or in a second independent patient cohort. Hence, the authors caution about the robustness of these data based on the low number of post mortem samples serving as control.

Thus, two independent studies, using two different profiling methodologies (microarray and sequencing) consistently detect a reduced expression of miR-184 in patients with mTLE + HS. However, the discrepancy in the total number of significantly dysregulated miRNAs identified in both studies could be related to the heterogeneity of the human samples. This is furthermore illustrated by the PCA plot, which showed a wide spread of human samples from both subgroups in our study as well as in other studies^19^. Another possibility for the observed discrepancy in the miRNA pattern could be related to potential sources for bias introduced by next generation sequencing (PCR amplification, reverse transcription, etc) or array based platforms (probe design, hybridization artifacts etc.). Additionally, other sources of discrepancies such as tissue quality, sample number, RNA extraction methods or data processing/analysis cannot be excluded (for review see ref. 28).

Astrogliosis and neuronal death are key pathological hallmarks of HS and a large body of evidence indicates activation of the innate immune system in the hippocampi of these patients^10^. The number of activated microglia cells is increased by a factor of ten in mTLE + HS patients’ hippocampi compared to control autopsy tissue^29^. Activated microglial cells and astrocytes contribute to the inflammatory response by secreting pro-inflammatory cytokines such as IL-1β, IL-6 and TNF-α^25^,^30^,^31^,^32^. Interestingly, several recent studies describe involvement of miRNAs in the regulation of inflammatory pathways identified in mTLE, for example miR146a^33^,^34^. Therefore, we evaluated the potential role of miR-184 in microglial activation. In our study we observe that overexpression of miR-184 is decreasing the activation of primary murine microglial cells which are known to be key players in neuroinflammatory processes. It is therefore tempting to speculate that the absence of miR-184 in mTLE patients with HS could lead to the more pronounced inflammation observed in those patients. Consistently with our study, a previous report indicated that miRNAs can have an profound effect on microglial activity during disease^35^. Furthermore, the release of pro-inflammatory cytokines, as described in the hippocampus of rats^36^, might also contribute to the increased neuronal loss observed in mTLE + HS patients. Interestingly, neuronal injury in the hippocampus is more pronounced when a combination of IL-6, TNF-α and IL-1β are elevated in rats post SE^37^. Although the molecular mechanism of miR-184 and the exact cellular pathways involved are currently unknown it is plausible that miR-184 could act as a negative regulator in microglial cells interfering with the inflammatory processes induced by seizures in the brain. Interestingly, miR-184 targets several genes involved in immune response and apoptosis that are differentially expressed in mTLE + HS patients. Although not all of these genes might be directly modulated by miR-184, it is plausible that miR-184 by ^2^inhibiting translation of some target genes might contribute to the regulation of these processes. However, the exact molecular mechanism and the entire set of target genes need to be investigated further.

In order to evaluate the potential contribution of altered miR-184 to the characteristic hippocampal neuronal cell loss in mTLE + HS patients, we overexpressed miR-184 in murine primary neurons. Previous *in vivo* studies did indicate that miR-184 could play a protective role in neuronal death as demonstrated in a mouse seizure preconditioning model^21^. In this study, miR-184 expression was increased upon preconditioning in pyramidal neurons of the CA1 and CA3 region of the hippocampus leading to reduced seizure induced neuronal death^21^. In our *in vitro* assay miR-184 overexpression itself did not positively affect cell viability of murine primary neurons. Moreover, our results are consistent with the clinical observations in mTLE patients; higher miR-184 expression is observed in mTLE -HS patients showing moderate neuronal death compared to mTLE + HS patients with more pronounced and characteristic neuronal cell loss and low miR-184 expression.

However previous findings also suggest that miR-184 inhibits cell proliferation via targeting AKT2 *in vitro* in neuroblastoma cell lines and *in vivo* in tumor patient samples^38^. Similarly, using a neuronal cell line (murine neuroblastoma cell line N1E-115), we observed a moderate dose dependent reduction of cell viability and an induction of caspase 3/7 activity after overexpression of miR-184 (Supplementary Figure S7). We speculate that this difference might be caused by mechanisms relevant for dividing neuroblastoma cells versus post mitotic neurons. However, to strengthen this hypothesis more studies are required to delineate the function of miR-184 on neuronal viability. Overall, based on available data it cannot be ruled out that a decreased expression of miR-184 might contribute to the more pronounced neuronal death observed in mTLE + HS patients. Nevertheless it is plausible that the decreased miR-184 expression observed in mTLE + HS patients is a consequence and not the cause of the more pronounced neuronal death observed in these patients when compared to mTLE -HS.

Astrogliosis is a common feature observed in epilepsy and contributes to the inflammatory response. Several miRNAs have been described to modulate inflammatory responses in astrocytes^34^,^36^ and astrogliosis^39^. In order to evaluate a potential influence of miR-184 on astrocytes, we overexpressed the miRNA in primary murine astrocytes and evaluated cell viability for several days. No impact on astrocyte viability was observed *in vitro*, suggesting that miR-184 expression levels do not directly modulate astrocyte proliferation. Indeed, astrogliosis is observed in both mTLE -HS patients and mTLE + HS patients suggesting that miR-184 is not likely to directly modulate astrogliosis.

In conclusion, this study identified miR-184 being dysregulated between mTLE + HS and mTLE -HS patients. Based on our *in vitro* data miR-184 could act as a potential modulator of the inflammatory processes occurring in mTLE patients by modulating the activation state of the microglial cells and thereby reducing cytokine release. Furthermore, no direct protective role of miR-184 in neurons and no direct modulation of astrocyte viability were detectable. Further investigation of the molecular targets of miR-184 in the hippocampal cell population could yield important information to delineate molecular pathways to strengthen our understanding of TLE and ultimately for the development of new therapies modulating the inflammatory response and consequently neuronal death in the brain of mTLE patients.

## Methods

### miRNA expression profiling in human brain samples

Human brain tissue was derived from pharmacoresistant mTLE patients undergoing surgery at the University Clinic Bonn. Patient selection was based on clinical evaluation at the University Clinic including EEG monitoring and MRI. All procedures were approved by and in accordance with the clinical Ethics Committee of the University Clinic Bonn. Informed consent for hippocampal resection as well as the usage of the tissue and information for research purposes was obtained from all patients in advance of the surgery. Immediately after en bloc resection the hippocampal tissue was cut along its longitudinal axis dividing it into hippocampal head and hippocampal body. The hippocampal body was fixed in 4% paraformaldehyde and embedded in paraffin for neuropathological analysis. The hippocampal head was snap frozen in dry ice for miRNA expression profiling. The frozen samples were stored at −80 °C. The hippocampal tissue was neuropathologically evaluated to assess the degree of hippocampal sclerosis (HS) using the Wyler classification whenever possible^8^. The range of histopathological analysis and immunohistochemistry depended on neuropathological findings. The standard panel included at least hematoxylin and eosin (H&E) staining and immunohistochemistry with antibodies against neuronal nuclear specific protein (NeuN, Millipore, USA) and glial fibrillary acid protein (GFAP, DakoCytomation, Denmark). Table 1 summarizes the number and clinical data of all patients included in this study. For miRNA expression analysis the hippocampal samples were divided into a group consisting of mTLE patients without HS (mTLE -HS, n = 10) and mTLE patients group showing HS (mTLE + HS, n = 14).

For RNA isolation from all specimens 25 μm sections were cut on a cryostat collecting 15 mg of tissue per sample. The RNA was isolated using mirVana miRNA isolation Kit according to the manufacturer’s procedure (Life Technologies). RNA concentration and quality were determined using NanoDrop and Agilent Bioanalyzer. All samples included into the miRNA expression profiling study had a RIN number (RNA quality) of at least 7.0.

The miRNA profiling was performed using Illumina deep sequencing service of Vertis Biotechnologie AG. 6 μg of total RNA per sample was used for the generation of cDNA pools. The miRNA sequencing was conducted using the Illumina Genome Analyzer II with 35 bp read length and 7.5 million reads per sample on average.

### Differential expression analysis and miR-184 target genes

Analysis of the sequencing data was performed by miR-Intess small RNA analysis pipeline (InteRNA Technologies B.V., Netherlands). Reads were pre-processed to trim the adapter sequences and mapped against the human genome assembly GRCh37. Annotations of the mapped loci were retrieved from Ensemble database (v. 65) and from miRBase (v. 18) and aligned reads were classified according to these annotations. Prediction of novel miRNA candidates was performed by miR-Intess as described previously^40^. Differentially expressed microRNAs were identified using the Bioconductor edgeR package. Data were normalized by applying the TMM (weighted trimmed mean of M-values) method and differential expression was assessed an exact test for the negative binomial distribution^41^. Only known microRNAs detected in at least 10 samples were taken into account. Correction for multiple testing was done according to Benjamini and Hochberg^42^. MicroRNA with a false discovery rate (FDR) lower than 0.01 were considered as significantly differentially expressed.

Potential target genes for miR-184 and biological pathways in which they are involved were identified using the MetaCore^TM^ software suite (http:// thomsonreuters.com/site/systems-biology). MetaCore^TM^ is an integrated software suite for functional analysis of Next Generation Sequencing, CNV, microarray, metabolic, SAGE, proteomics, siRNA, microRNA. MetaCoreTM is a manually-curated database containing microRNA targets based on experimental validation from literature or target prediction algorithms.

### Quantitative PCR (RT-qPCR)

For miRNA expression reverse transcription (RT) was carried out with the Universal cDNA Synthesis kit from Exiqon using 20 ng of total RNA. qPCR was performed using the ExiLENT SYBR^®^ Green master mix and miRCURY LNA Universal RT microRNA PCR primer sets following manufacturer’s recommendations (Exiqon INC., USA). Briefly, qPCR reactions were performed in a 384 well plate using 5 μl MasterMix and 1 μl primer for each miRNA and 4 μl 1:80 cDNA per well. To identify suitable reference miRNAs 5 stably expressed miRNAs were selected based on NGS data. In a pre-screen the two most stably expressed miRNA were identified using the geNorm + module in the qBase + software^43^. Each sample was then run in triplicates for miR-184 as well as for two reference miRNAs (miR-125a-5p and miR-191-5p).

For target gene expression analysis cDNA synthesis was performed with High Capacity cDNA RT Kit (Life Technologies) and inventoried TaqMan Gene expression assays were used for qPCR together with TaqMan GenEx Master Mix (Life Technologies) following manufacturer’s instructions. The stably expressed human GAPDH gene was identified using the geNorm + module in qbase + and used as reference genes for normalization^43^.Normalized relative expression levels for miRNA and mRNA were calculated using the qbase + software^44^ (Biogazelle NV, Zwijnaarde, Belgium).

### *In situ* hybridisation

Sections were incubated in Xylol for 2 hours and then 5 min in 99.9%, 96% and 70% ethanol respectively. Sections were rinsed with TBST (TBS + 0.1% Tween 20) and then incubated with Proteinase K (10 μg/mL) for 7 min at 37 °C. After washing the sections with TBS they were treated with 4% PFA for 10 min. Sections were washed and treated with 0.2% Glycin in TBS, rinsed again with TBS and acetylated for 30 min with triethanolamin/acetic anhydrid. Slides were then rinsed in hybridization buffer (5× SSC, 50% formamide and 5× Denhardt’s, 250 μg/ml yeast t-RNA (Sigma) 2% blocking reagent (Roche), 0.1% Tween 20) for 2 h at RT. Probes (4 pmol) were incubated in hybridization buffer overnight at Tm −20 °C (Tm provided by Exiqon) in a humidified chamber. miR-124 served as positive control for hybridization and miR-scr served as negative control. All probes were 5′-3′-digoxigenin-labeled. The following day sections were washed in 5× SSC to remove cover slides and then washed with washing buffer 1 (50% formamide, 1xSSC, 0.1% Tween 20), for 30 min at Tm −20 °C. Then sections were rinsed in washing buffer 2 (0.2 × SSC) for 15 min at room temperature and TBST for 5 min. Sections were incubated with blocking solution (for 1 h at RT and then incubated with Anti-DIG-Fab POD antibody (1:200, Roche) for 1 h at RT. After rinsing sections with TBST they were treated with TSAPlus Cy3 working solution (PerkinElmer) for 10 min. Afterwards sections were washed again with TBS. As control sections were co-stained with anti-GFAP antibody (Millipore) as follows: after blocking (2% BSA, 0.1% Tween 20) sections were incubated with anti-GFAP (Millipore) overnight. The following day sections were washed in PBS and incubated in blocking solution for 20 min at RT. Sections were incubated with Alexa Fluor 488 labelled antibody (Life technologies) for 2 h and washed with PBS.

### Experimental Animals

Pregnant female C57BL/6 mice were purchased from Charles River Laboratories (Sulzfeld, Germany). Mice were kept individually under standard housing conditions (12 h dark-light cycle; food and water available ad libitum). All experiments were carried out in accordance and approved by the LANUV (Landesamt für Natur, Umwelt und Verbraucherschutz Nordrhein-Westfalen) and state regulation for research with animals.

### Primary Microglia Isolation and Culture Conditions

Brains of P0-3 pups from C57BL/6 mice were used to isolate microglial cells. A single cell suspension was prepared using papain based neural tissue dissociation protocol (Miltenyi Biotec, 130-092-628). Primary microglia were isolated using CD11b Microbeads (Miltenyi Biotec GmbH, 130-093-634) according to the manufacturer’s instruction. The purity of the isolated cells was determined by staining with fluorescently labelled antibodies APC-CD45 and FITC-CD11b (Miltenyi Biotec, 130-091-811 and 130-081-201) and analyzed by Flow Cytometry (purity was approximately 90%). Microglia cells were re-suspended in DMEM/F12 (Gibco, 11320-074) supplemented with 10% FBS (Gibco, 10082139), 0.1% nonessential amino acids (Gibco, 11140-050), 0.1% GlutaMAX (Gibco, 35050-038) and 1% Penicillin/Streptomycin and plated into 96 well plates (2×10^4 ^cells/well) for cytokine release using Meso Scale Discovery (MSD) or into 12-well plates for RNA isolation.

### Microglia Activation Assay

Transfection with miRNA-mimic was performed at DIV7. 30 nM of miRNA-mimic (Life Technologies, mirVanaTM miRNA Mimics) were complexed with 0.5 μl Lipofectamine RNAiMAX Transfection Reagent (Life Technologies, 13778) according to manufacturer’s instructions. MiRNA sequences used for transfection were: mir-184: 5′-UGGACGGAGAACUGAUAAGGGU-3′ (Life Technologies, MC10207) and the negative control miRNA-mimic (Life Technologies, 4464058). Two days later cells were incubated with 4 μg/mL LPS (Enzo Life Science, 581-007-LOOZ) and 10 ng/mL IFN-γ (Miltenyi Biotec, 130-096-872) for 24 h. Cytokine release was measured following manufacturer’s instructions in the cell culture supernatant from 24 h stimulated cultures using a mouse pro-inflammatory 7-plex ultra-sensitive kit (K15012C) from Meso Scale Discovery (MSD; Gaithersburg, USA).

### Primary Neuron Isolation and ATP-Assay

Primary murine neurons were isolated using neuronal isolation kit (Miltenyi Biotec, 130-098-754) according to the manufacturer’s instructions. Briefly, the brains of P0-1 pups from pregnant C57BL/6 mice were isolated. Papain based neural tissue dissociation protocol was used to obtain a single cell suspension (Miltenyi Biotec, 130-092-628). Neurons were cultured in Neurobasal A medium (Invitrogen, 10888-022) supplemented with 0,1% GlutaMAX (Gibco, 35050-038), 1% B27 (Gibco 17504-044), 1% N2 supplement (Gibco) and 1% Penicillin/Streptomycin. Cells were plated into 96 well plates (4×10^4 ^cells/well) for ATP-Assay or into 12 well plates (5×10^5 ^cells/well) for RNA isolation. On DIV 4 cells were transfected with 30 nM of miRNA-184-mimic (Life Technologies, mirVanaTM miRNA Mimics, MC10207, sequence: 5′-UGGACGGAGAACUGAUAAGGGU-3′) and the corresponding scr-control (Life Technologies, 4464058) using 0.5 μl Lipofectamine RNAiMAX Transfection Reagent (Life Technologies, 13778) according to manufacturer’s instructions. To determine cell proliferation/cytotoxicity ATPlite Assay (ATPlite 1step, PerkinElmer, 6016731) was performed 72 h after transfection.

### Primary Astrocyte Cell Culture, Transfection and Viability assay

Primary astrocytes were prepared from postnatal mouse brains (P0-P3) using mechanical dissociation method modified from previous studies^45^,^46^. Briefly, the pup was decapitated and the brain was separated. Cerebellum and olfactory bulb were removed and leptomeninges and peripheral blood vessels were gently removed from the forebrain. Subsequently, the forebrain was dissociated by pipetting with a 1-ml Pasteur pipette. The yielded cell suspension was gently passed through a 40 μm cell strainer (BD, 352340), followed by centrifugation. The supernatant was removed and the pellet re-suspended in 5 ml DMEM GlutaMax (Gibco, 31966-021) supplemented with 10% FBS (Gibco, 10082139); 1% Penicillin/Streptomycin (Life Technologies, 15140-122), and transferred onto a 25-cm^2^ flask (Greiner bio-one, 690940). Cell cultures were maintained at 37 °C with 5% CO_2_. One day after preparation, cell debris was removed by washing twice with PBS and addition of 5 ml fresh culture medium. The medium was renewed every three days. After 10–14 days, a confluent layer of cells were generated which contain glial progenitor cells and microglia growing on the monolayer. The latter were separated mechanically by agitation and were removed with the PBS wash solution. At least 90–95% of these cells in culture were type I astrocytes^47^. For miR-184 overexpression 30 nM of miRNA-184-mimic (Life Technologies, mirVanaTM miRNA Mimics, MC10207, sequence: 5′-UGGACGGAGAACUGAUAAGGGU-3′) were complexed with 0.5 μl Lipofectamine RNAiMAX Transfection Reagent (Life Technologies, 13778) according to manufacturer’s instructions. As negative control for miRNA-mimic transfection the control miRNA-mimic (Life Technologies, 4464058) was used. To determine cell viability the colorimetric MTT metabolic activity assay was used. Astrocytes (5000 cells/well) were cultured in a 96 well plate at 37 °C with 5% CO_2_. Astrocytes cultured with medium and lipofectamine were considered as control group. 5 mg/ml MTT was added to the medium. After 2 h incubation at 37 °C the formazan crystals were dissolved in 50 μl dimethyl sulfoxide (DMSO, Sigma, 472301). The absorbance intensity measured by a microplate reader (PerkinElmer, EnSpire Mulimode Plate Readers; USA) at 540 nm with a reference wavelength of 600 nm. All experiments were performed in quadruplicate and the cell viability was measured at 2-days intervals from day 0 to day 11 after mimic miRNA transfection.

### RNA Extraction and Quality Determination

Total RNA was extracted from primary microglia, neurons and astrocytes using mirVana miRNA isolation kit (Life Technologies, AM1560) according to the manufacturer’s protocol. The RNA concentration and purity were determined using a NanoDropTM ND-2000 spectrophotometer (Thermo Scientific, Waltham, USA) in all samples. Moreover, RNA integrity was analysed using the Agilent 2100 Bioanalyzer (Agilent Technologies, Santa Clara, USA).

### Luciferase Reporter Gene Assay

HELA cells were co-transfected in a 96 well plate using GenMute™ siRNA Transfection Reagent with modified reporter vector (Genecopoeia pEZX-FR02) and 40pmol of miR-184 mimic (Life Technologies, mirVanaTM miRNA Mimics, MC10207) or miRNA mimic control (miR-scr, Life Technologies, 4464058). Forty-eight hours after transfection luciferase activities were measured using Luc-Pair Duo-Luciferase Assay from Genecopoeia, according to manufacturer’s instructions. All experiments were performed twice in triplicate. For each sample the Firefly luciferase activity was normalize to the Renilla luciferase activity used as a control to standardize for transfection efficiency.

### Statistical Analysis

All data were normally distributed and presented as mean values ± s.e. or s.d. as specified. Statistical differences between two groups were evaluated by two tailed unpaired Student’s t-test or with a two-sided Mann Whitney test with t approximation. In the case of multiple mean comparisons one-way analysis of variance (ANOVA) was used (Gnumeric 1.12.12; SPSS Statistics 19, IBM, USA). P-values < 0.05 were regarded as significant.

## Additional Information

**How to cite this article**: Danis, B. *et al*. Differential expression of miR-184 in temporal lobe epilepsy patients with and without hippocampal sclerosis – Influence on microglial function. *Sci. Rep.* **6**, 33943; doi: 10.1038/srep33943 (2016).

## Supplementary Material

Supplementary Table S1

Supplementary Information

## Figures and Tables

**Table 1:** Clinical data of mTLE patients with (HS) and without hippocampal sclerosis (nonHS).

Sample name	Disease duration (years)	Age (years)	gender	Age of onset (years)	AEDs	Wyler Score	histology	pharmaco-resistant
HS01	40	43	F	3	cbz, oxc, pb/prm, ltg, tpm, lev, lcm, vgb	atypical HS	astrogliosis, neuronal loss	yes
HS02	20	33	M	13	cbz, vpa, pht, ltg, lev	III	astrogliosis, neuronal loss	yes
HS03	10	23	F	13	vpa, gbp, ltg, lev, lcm	n.d.	astrogliosis, neuronal loss	yes
HS04	37	42	M	5	cbz, oxc, vpa, pb/prm, ltg, lev, benzos, clo	atypical HS	astrogliosis, neuronal loss, activated microglia	yes
HS05	35	46	M	11	cbz, pht, gbp, lev, lcm, benzos, dzp	IV	astrogliosis, neuronal loss, activated microglia, postencephalitic cicatrice	n.m.
HS06	33	48	M	15	cbz, vpa, pht, pb/prm, ltg, tpm, lev, vgb	III	astrogliosis, neuronal loss, activated microglia	yes
HS07	23	57	M	34	cbz, oxc, lev, pgb	III	astrogliosis, neuronal loss, activated microglia, lipome	yes
HS08	44	47	M	3	cbz, vpa, pht, ltg, lev, zon, benzos, clo	IV	astrogliosis, neuronal loss, activated microglia	n.m.
HS09	18	48	F	30	cbz, oxc, vpa, gbp, ltg, lev, pgb, benzos	IV	astrogliosis, neuronal loss, activated microglia	yes
HS10	43	46	F	3	cbz, oxc, vpa, lev	III	astrogliosis, neuronal loss	n.m.
HS11	20	33	F	13	cbz, vpa, ltg, lev, pgb, zon, lcm	IV	astrogliosis, neuronal loss	yes
HS12	22	23	F	1	oxc, vpa, pb/prm, ltg, tpm, lev, lcm	IV	astrogliosis, neuronal loss, activated microglia	yes
HS13	9	18	M	9	cbz, oxc, ltg, lev	IV	astrogliosis, neuronal loss, activated microglia	n.m.
HS14	2	3	M	1	cbz, lev	n.d.	astrogliosis, activated microglia, ganglioglioma	n.m.
nonHS01	26	55	F	29	cbz, vpa, gbp, lev, benzos	—	astrogliosis, neuronal loss, infarct	yes
nonHS02	8	51	M	43	oxc, ltg, lev, zon, lcm	—	astrogliosis, activated microglia	n.m.
nonHS03	13	15	M	2	oxc, lev, st	—	astrogliosis, activated microglia	n.m.
nonHS04	28	31	F	3	cbz, oxc, vpa, pht, pb/prm, gbp, ltg, tpm, lev, pgb, zon	—	astrogliosis, activated microglia	yes
nonHS05	18	45	M	27	vpa, tpm, lev	—	astrogliosis, activated microglia, dysembryoplastic, neuroepithelial tumour (DNT)	yes
nonHS06	2	45	F	43	vpa, gbp, ltg, lev	—	astrogliosis	n.m.
nonHS07	16	24	F	8	cbz, oxc, esl, lev, pgb	—	activated microglia	n.m.
nonHS08	7	8	F	1	vpa, ltg, lev	—	astrogliosis, activated microglia	yes
nonHS09	27	44	F	17	cbz, oxc, esl, ltg, tpm, lev, pgb, zon, lcm, benzos	—	astrogliosis, neuronal loss	yes
nonHS10	18	27	M	9	cbz, oxc, vpa, ltg, tpm, lev, zon	—	ganglioglioma	yes

*AED* anti-epileptic drug, *HS* hippocampal sclerosis, *F* female, *M* male, *n.d.* not determinable, *n.m.* not mentioned, *cbz* carbamazepine, *oxc* oxcarbazepine, *pb/prm* phenobarbital/primidon, *ltg* lamotrigine, *tpm* topiramat, *lev* levetiracetam, *lcm* lacosamid, *vgb* vigabatrin, *vpa* valproinic acid, *pht* phenytoin, *gbp* gabapentin, *benzos* benzodiazepine, *clo* clobazam, *dzp* diazepam, *zon* zonisamid, *pgb* pregabaline, *st* sultian, *esl* eslicarbazepine acetat.
