# Supplementary material for: Differential expression of miR-184 in temporal lobe epilepsy patients with and without hippocampal sclerosis – Influence on microglial function
Source: Sci Rep. 2016 Sep 26;6:33943. doi: 10.1038/srep33943 (PMC5036198; doi:10.1038/srep33943)
Supplement: Supplementary Information [file srep33943-s2.pdf]

Differential expression of miR-184 in temporal lobe epilepsy patients with and without hippocampal sclerosis – Influence on microglial function.

Bénédicte Danis[2*], Marijke van Rikxoort[1*], Anita Kretschmann[1], Jiong Zhang[1], Patrice Godard[3], Lidija Andonovic[1], Franziska Siegel[1], Pitt Niehusmann[4], Etienne Hanon[2], Daniel Delev[5], Marec von Lehe[6], Rafal M. Kaminski[2], Alexander Pfeifer[1§], Patrik Foerch[2§]

[1]Institute of Pharmacology and Toxicology, University of Bonn, Sigmund-Freud-Str. 25, 53127 Bonn, Germany

[2]UCB Pharma S. A., Chemin du Foriest, B - 1420 Braine-l'Alleud, Belgium

[3]Thomson Reuters, IP & Science, 5901 Priestly Dr., #200, Carlsbad, CA 92008, USA

[4]Deptartment of Neuro- / Pathology, Oslo University Hospital, Sognsvannsveien 20, 0372 Oslo, Norway

[5]Neurosurgery, University of Bonn, Sigmund-Freud-Str. 25, 53127 Bonn, Germany

[6]Neurosurgery, Universitätsklinikum Knappschaftskrankenhaus Bochum, Bochum, Germany

* these authors contributed equally to this work

[§] correspondence should be addressed to Patrik Foerch and Alexander Pfeifer

Email: patrik.foerch@ucb.com and alexander.pfeifer@uni-bonn.de

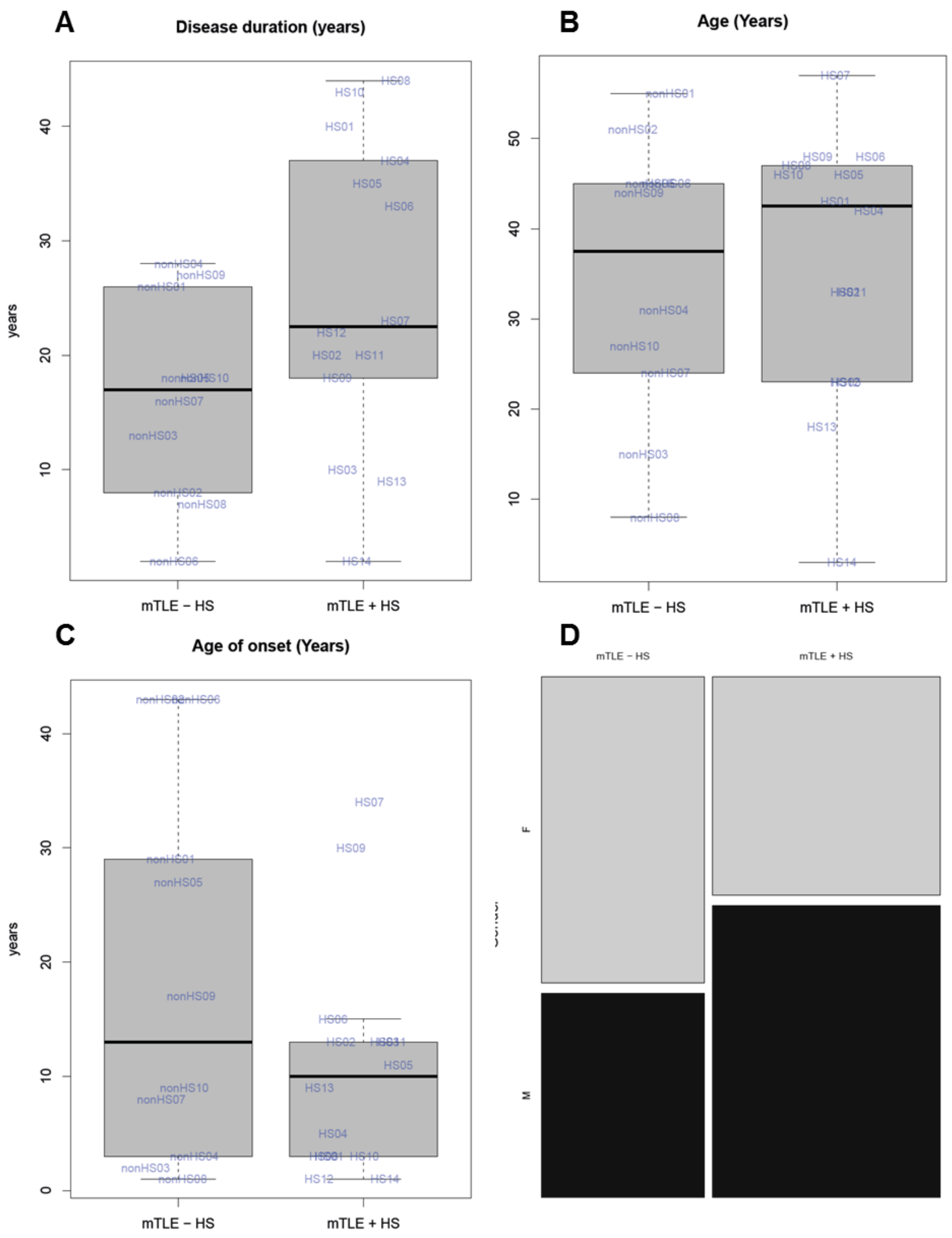

Supplementary Figure S1: Comparison of several cofactors values in the 2 patient cohorts. A) Disease duration. B) Age. C) Age of onset. D) Gender. No significant difference was observed between the 2 patient cohorts.

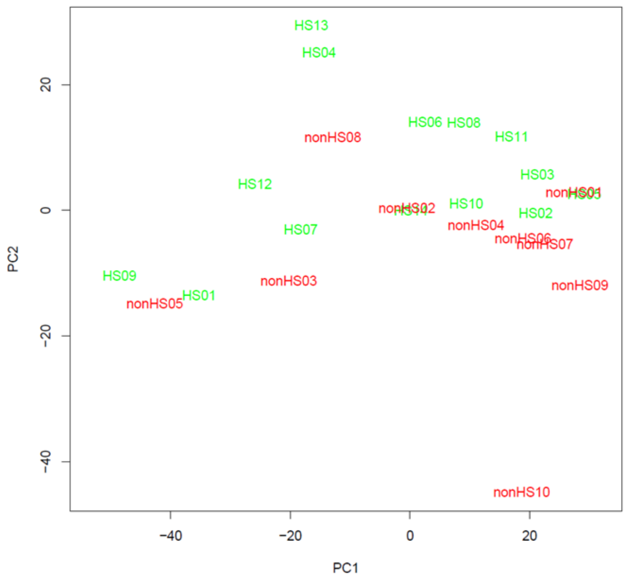

Supplementary Figure S2: Principal component analysis (PCA) of microRNA expression data. In red mTLE -HS patients and in green mTLE +HS patients.

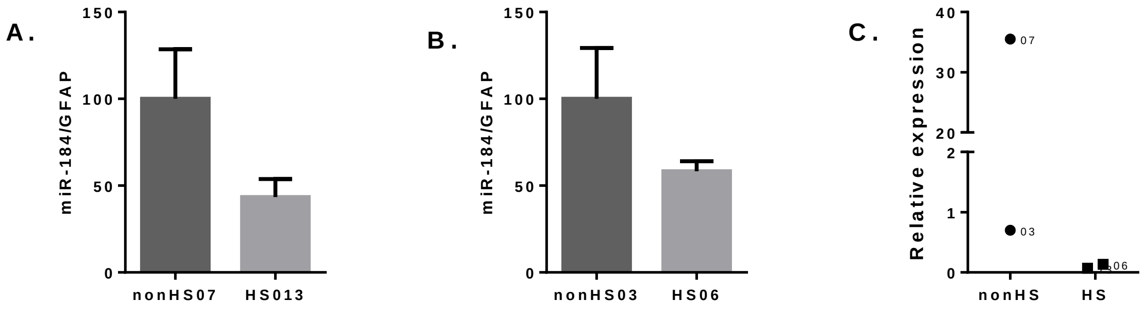

Supplementary Figure S3: Quantification of in situ hybridization analysis for miR-184 in mTLE patients. Double-labelled miR-184/GFAP cells were quantified blinded from three pictures per patient by two independent operators. Data represent the mean +/- standard error of the three pictures with the nonHS patient set at 100%. A high miR-184 expressing nonHS patient (A) versus are low miR-184 expressing nonHS patient are represented (B), miR-184 relative expression measured by RT-qPCR is shown for all 4 patients in C.

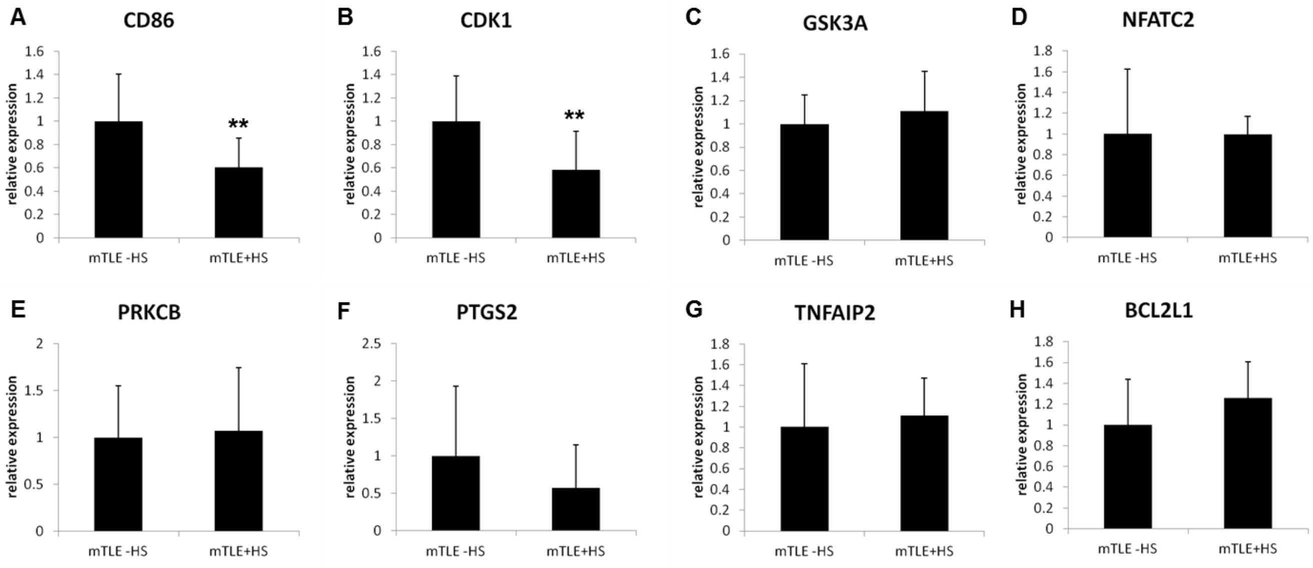

Supplementary Figure S4: Expression of miR-184 target genes in hippocampal tissue. The expression of selected target genes of miR-184 was investigated by qPCR in samples of mTLE -HS and mTLE +HS patients. Results show means of all samples and technical triplicates. Error bars represent s.d. **p<0.05, *p<0.1.

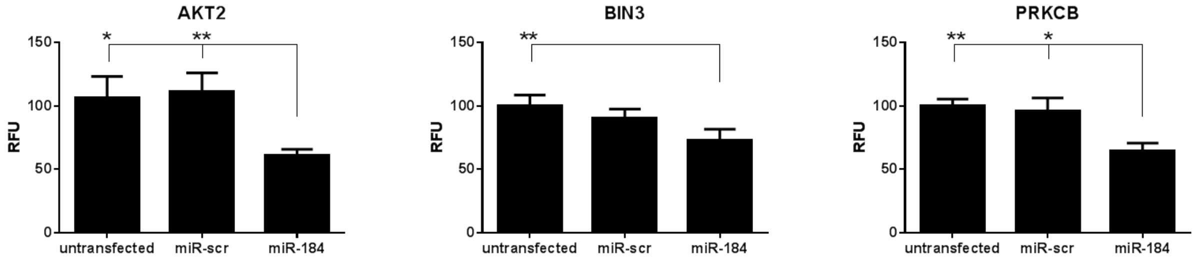

Supplementary Figure S5: Evaluation of miR-184 3'UTR targeting of the predicted targets in a luciferase reporter gene assay. HELA cells were co-transfected with reporter vector and 40pmol of miR-184 mimic or miRNA mimic control (miR-scr). Forty-eight hours after transfection luciferase activities were measured. Data represent the relative fluorescence units (RFU) of firefly luciferase activity normalize to renilla luciferase expression. Mean activities +/- s.d. of two independent experiments in triplicate are shown. **p<0.05, *<0.1.

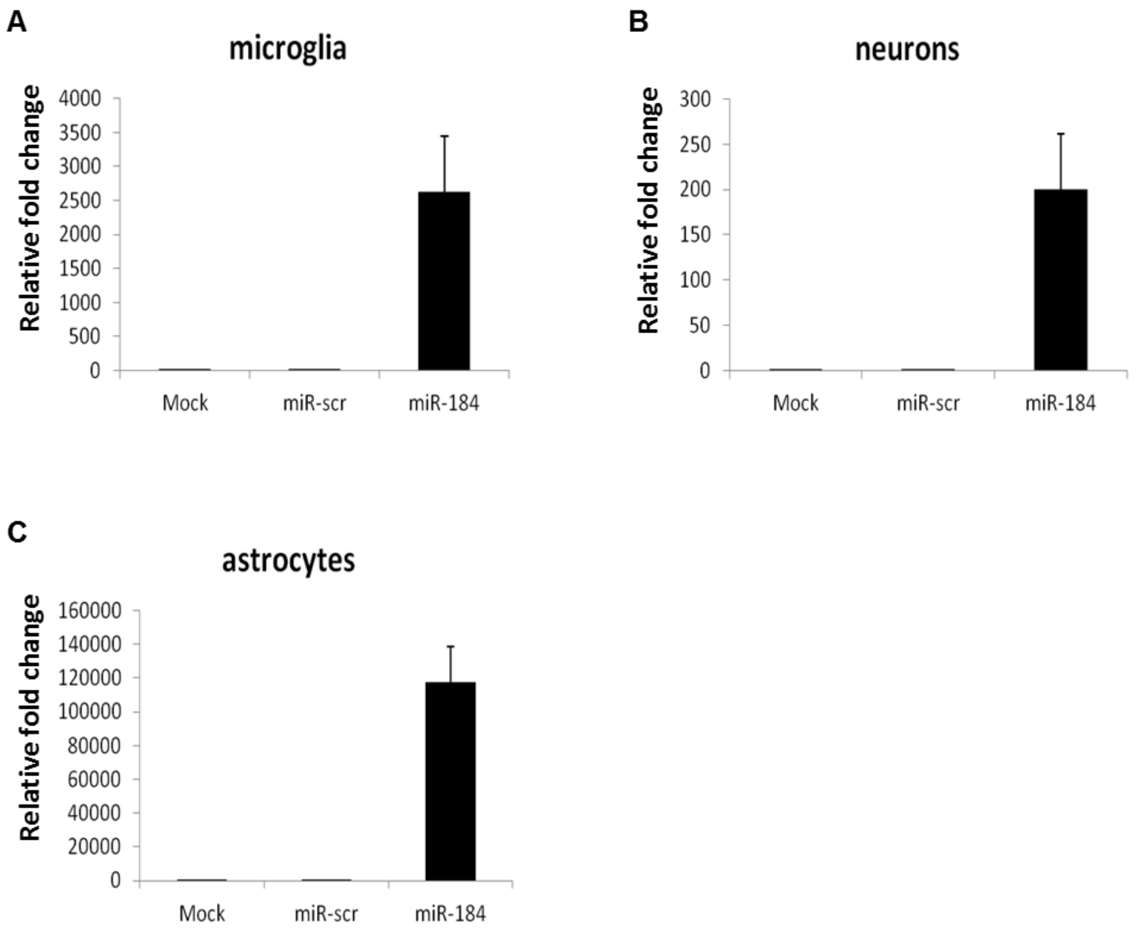

Supplementary Figure S6: Overexpression levels of mir-184. Expression levels were determined by RT-qPCR in triplicates. Data represent mean +/- s.d. (A) mir-184 expression levels in primary murine microglial cells 72 hours after transfection. (B) mir-184 expression levels in primary murine neurons 72 hours after transfection. (C) mir-184 expression levels in primary murine astrocytes 72 hours after transfection.

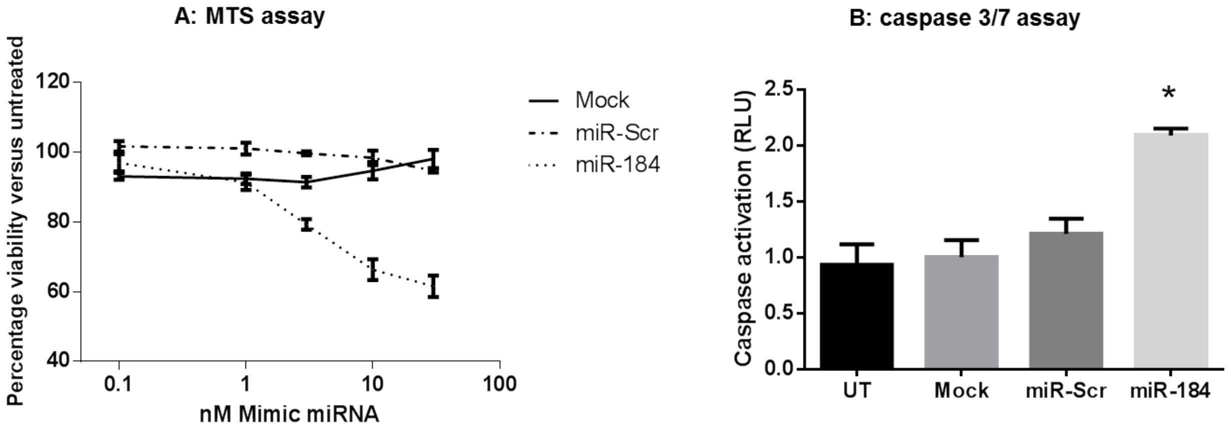

Supplementary Figure S7: Influence of miR-184 overexpression in a murine. neuroblastoma cell line. (A) Viability of the cells was measured using a MTS assay 48h after miRNA mimic transfection. Data represent the mean of triplicates +/- s.d. from one representative experiments, data was normalized to the untreated cells. (B) Caspase 3/7 activation assay 48h after miRNA mimic transduction. Bar graph represent the mean of 2 independent experiments +/- s.d. ($p<0.05$).
